# Misperception of body verticality in neurological disorders: A systematic review and meta‐analysis

**DOI:** 10.1002/brb3.3496

**Published:** 2024-04-30

**Authors:** Esteban Obrero‐Gaitán, David Fuentes‐Núñez, María Del Moral‐García, María del Carmen López‐Ruiz, Daniel Rodríguez‐Almagro, Rafael Lomas‐Vega

**Affiliations:** ^1^ Department of Health Sciences University of Jaen Jaen Spain; ^2^ Department of Nursing, Physiotherapy and Medicine University of Almeria Almeria Spain

**Keywords:** body verticality, nervous system diseases, stroke, subjective postural vertical

## Abstract

**Introduction:**

The internal representation of verticality could be disturbed when a lesion in the central nervous system (CNS) affects the centers where information from the vestibular, visual, and/or somatosensory systems, increasing the risk of falling.

**Objective:**

The aim was to evaluate the vestibular and somatosensory contribution to the verticality pattern in patients with stroke and other neurological disorders.

**Methods:**

A literature search was performed in PubMed, Scopus, Web of Science, and CINAHL databases. Cross‐sectional, case–control, and cohort studies comparing body verticality in patients with stroke or CNS diseases (CNSD) versus healthy controls were selected. Subjective postural vertical (SPV) in roll and pitch planes was used as the primary variable.

**Results:**

Ten studies reporting data from 390 subjects were included. The overall effect for CNSD patients showed a misperception of body verticality in roll (standardized mean difference [SMD] = 1.05; 95% confidence interval [CI] .84–1.25) and pitch planes (SMD = 1.03; 95% CI .51–1.55). In subgroup analyses, a high effect was observed in the perception of SPV both in roll and pitch planes in stroke (*p* = .002) and other CNSD (*p* < .001).

**Conclusion:**

These findings suggest a potential misperception of SPV in patients with stroke and other neurological disturbances. Patients with CNSD could present an alteration of vestibular and somatosensory contribution to verticality construction, particularly stroke patients with pusher syndrome (PS), followed by those with PS combined with hemineglect.

## Graphical abstract

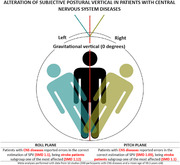

## 1 INTRODUCTION

Stroke is the pathology most frequently related to alterations in verticality perception (Baggio et al., 2016) and may present with postural alterations (Barra & Perennou, 2013) and a reduction in coordination associated with weakness and loss of unilateral sensitivity (Rodgers, 2013). In addition, stroke may involve other concomitant dysfunctions, such as pusher's syndrome (PS) (Karnath & Broetz, 2003), resulting in postural destabilization and consequent falls toward the affected side, or spatial neglect (SN) (Yang et al., 2012), leading to reduction of capacity to report upon contralesional (and also ipsilateral in some cases with moderate to severe SN) inputs and neglecting to explore the contralesional hemispace (Kerkhoff et al., 2009; Pérennou, 2006).

Verticality perception is achieved by a complex integration and modulation processes of the inputs coming from vestibular, visual, and somatosensory systems (Bronstein, 2016) into the central nervous system (CNS) (Martín Sanz et al., 2004). The correct estimation at the central level of verticality allows posture organization with respect to the gravitational environment (José Luvizutto et al., 2020). Vestibular, which provides three‐dimensional spatial perception (Bronstein, 2016; Dieterich & Brandt, 2019), and visual systems, which collect information about the structure and three‐dimensional disposition of the environment, play a primary role in this process. The information collected from the above systems is jointly integrated with the position and muscular tension of each body segment, provided by the somatosensory system (Lopez et al., 2007), at central level to build an internal verticality representation (Dieterich & Brandt, 2019) that is continuously updated (Barra & Perennou, 2013).

The internal representation of verticality could be disturbed when a lesion in the CNS affects the centers where information from the vestibular, visual, and/or somatosensory systems is processed and integrated representation (Dieterich & Brandt, 2019). After stroke, the integration of stimuli or their processing may be damaged, thus disturbing the internal representation of verticality. Some previous studies have suggested the presence of an alteration of verticality after stroke (Baggio et al., 2016), both in subjects with PS and SN (Embrechts et al., 2023). In this line, patients with pushing behavior have manifested ipsilesional subjective postural vertical (SPV) tilts that are as severe as the severity of the behavior, as well as significant difficulties in estimating verticality in both pitch and roll planes (Bergmann et al., 2020). In addition, a stroke could affect certain networks that process three‐dimensional spatial information, resulting in SN and a disturbed perception of verticality, simultaneously (Kerkhoff et al., 2009).

The different parameters of the internal representation of verticality can be estimated through evaluating verticality perception, taking as reference visual (subjective visual vertical test), haptic (subjective haptic vertical test), and postural information (SPV test) (Dakin & Rosenberg, 2018). More specifically, the SPV test enable to estimate the contribution of the vestibular and somatosensory systems to the construction to the internal verticality model of each subject, due to it is performed without the contribution of the visual information (Dakin & Rosenberg, 2018).

Although numerous studies have been performed to determine the visual contribution to verticality patterns in subjects with neurological (Molina et al., 2019) or spinal diseases (Obrero‐Gaitán et al., 2020) a small but emerging interest in the assessment of the contribution of vestibular and somatosensory systems to verticality construction in patients with neurological pathology is occurring. Following this interesting research, the present systematic review and meta‐analysis has set as the primary objective to detect, collect, and perform a quantitative synthesis of the best available evidence regarding the vestibular and somatosensory contribution to the verticality central pattern in patients with stroke and other neurological problems using SPV assessment. It also investigated the possibility of detecting differences in SPV perception as a function of pathology.

## 2 METHODS

### 2.1 Protocol review

This systematic review and meta‐analysis was performed following the recommendations of the updated version of the Preferred Reporting Items for Systematic Reviews and Meta‐Analyses (PRISMA) statement (Page et al., 2021) and the guidelines of the Meta‐Analysis of Observational Studies in Epidemiology (MOOSE) group (Stroup et al., 2000). In addition, this review was previously registered in PROSPERO (CRD42021286244).

### 2.2 Data sources and search strategy

Two authors (D.F.‐N. and M.M.‐G.), independently performed a bibliographic search in PubMed Medline, SCOPUS, Web of Science (WOS), and CINAHL through April, 2023. Other sources, such as gray literature, meeting abstracts, guidelines, previous reviews, or the reference lists from retrieved full‐text studies, were screened to identify potential studies to be included. The keywords used, based on Medical Subjects Headings (MeSH) and previously published studies, were “SPV,” “CNS diseases (CNSD)” or “stroke.” These keywords and entry terms were combined employing the appropriate tags and the Boolean operators “and”/“or.” Filters related to language and publication date were not used. This stage was supervised by an expert author in the bibliographical search (E.O.‐G.). Search strategy used in each database is summarized in Table 1.

**TABLE 1:** Bibliographic search strategy.

Databases	Search strategy
PubMed Medline	(Nervous system diseases[mh] OR nervous system diseases[tiab] OR central nervous system diseases[mh] OR central nervous system diseases[tiab] OR stroke[mh] OR stroke[tiab] OR cerebrovascular accident[tiab]) AND (“postural vertical”[tiab] OR “subjective postural vertical”[tiab] OR “gravitational vertical”[tiab])
SCOPUS	(TITLE‐ABS‐KEY (“nervous system disease” OR “central nervous system disease” OR “stroke”) AND TITLE‐ABS‐KEY (“perception of verticality” OR “postural vertical” OR “subjective postural vertical” OR “gravitational vertical”)
Web of Science	TOPIC (*nervous system diseases* OR *central nervous system diseases* OR *stroke*) AND TOPIC (*postural vertical* OR *Subjective postural vertical* OR *gravitational vertical*)
CINAHL Complete	AB (nervous system diseases OR central nervous system diseases OR stroke) AND AB (postural vertical OR Subjective postural vertical OR gravitational vertical)

### 2.3 Study selection: inclusion and exclusion criteria

Two authors (D.F.‐N. and M.C.L.‐R.) independently reviewed the titles and abstracts of all references retrieved in the databases. If based on the title and abstract screening, one of these two authors selected a reference during this stage, it was examined in detail. Irrelevant studies were excluded based on screening of the title and abstract. All disagreements were resolved with a third expert author (D.R.‐A.).

To be included in this review, an article had to meet all the following inclusion criteria: (1) observational study (cross‐sectional, case–controls and cohort studies); (2) assessed the ability of patients with nervous system diseases (exposed group); (3) estimated the perception of SPV in the roll or pitch plane; (4) performed before therapeutic interventions; (5) compared cases to healthy controls; and (6) provided quantitative data to be integrated into the meta‐analysis. The following were exclusion criteria: (1) observational studies with only one group; (2) studies that did not report the mean or standard deviation of the SPV estimation; and (3) studies that did not obtain the absolute error mean and its standard deviation according to validated procedures (Higgins et al., 2011; Hozo et al., 2005).

### 2.4 Data extraction

Two authors (M.C.L.‐R. and D.F.‐N.) independently collected data from the studies included in the review using a Microsoft Excel standardized data collection form that we created. A third author (R.L.‐V.) was consulted to resolve any disagreements.

We extracted the following characteristics from each selected study: (1) overall study characteristics (authorship, publication date, and study design); (2) characteristics related to the sample (total sample size, number of groups, number of participants in exposed and nonexposed groups, age, gender sex); (3) characteristics related to pathology, type of specific pathology, and time since diagnosis); and (4) characteristics related to outcomes. The primary outcome measured was the perception of body verticality obtained in each group using the SPV test. In addition, we recorded whether SPV was used to assess body verticality in the roll or pitch plane. All data included in our analysis according to SPV were obtained without any therapeutic intervention in the case and healthy groups. For each group, we extracted the mean and standard deviation of the SPV measurements. However, if the study did not report standard deviation data, it was estimated following the guidelines of the Cochrane Handbook for Systematic Reviews of Interventions (Higgins & Green, 2011) and Hozo et al. (2005) using standard error, interquartile range, range, or confidence interval (CI) limits provided in each study.

### 2.5 Methodological quality assessment

Two authors (D.F.‐N. and D.R.‐A.) independently assessed the methodological quality of the studies included using the Newcastle–Ottawa scale (NOS) (Wells et al., 2000). Doubts and discrepancies were resolved by a third expert author (R.L.‐V.). The NOS is used to assess the methodological quality of observational studies exploring different domains, such as “selection of study groups” (maximum, 4 stars), “comparability of groups” (maximum, 2 stars), and “ascertainment of exposure/outcome” (maximum, 3 stars). The total score of a study can range from 0 (very low quality) to 9 (high quality). In addition, quality may be classified into ranges: low (score 1–3), medium (score 4–6), and high quality (score 7–9). Based on the Grading of Recommendations Assessment, Development and Evaluation (GRADE) system (Atkins et al., 2004), we used the GRADE checklist from Meader et al. (2014) to estimate the quality of evidence, integrating the imprecision, inaccuracy, and risk of publication bias. Inconsistency was evaluated using heterogeneity of findings in individual studies and imprecision through the number of included studies (large: >10 studies, medium: 5–10 studies, and small: <5 studies) and with the median sample size of each study (high: >300 subjects, medium: 100–300 subjects, and low: <100 subjects) (Higgins et al., 2003; Meader et al., 2014). The assessment of publication bias risk is detailed in the statistical analysis subsection.

### 2.6 Statistical analysis

Meta‐analysis was performed by two authors (E.O.‐G. and M.M.‐G.) using Comprehensive Meta‐Analysis 3.3.070 software (Biostat). We follow the recommendations of Cooper et al. (2009), employing a random‐effects model of DerSimonian and Laird (1986) to estimate the pooled effect with the aim of generalizing our findings. The pooled effect was estimated through Cohen's standardized mean difference (SMD) and its 95% CI (Cohen, 1977). SMD can be interpreted as small (SMD = .2), moderate (SMD = .5), or large (SMD > .8) (Faraone, 2008). Findings are displayed using forest plots. The risk of publication bias was assessed using visualization of the funnel plot (symmetric = low risk of publication bias or asymmetric = high risk of publication bias) and with the *p* value for Egger's test (*p* value <.1 indicates possible risk of publication bias) (Peters et al., 2006; Sterne & Egger, 2001). In addition, we used the trim‐and‐fill method of Duval and Tweedie (2000) to estimate the adjusted pooled effect, considering a possible publication bias (Shi & Lin, 2019). Heterogeneity was assessed using Cochran's *Q* test (Higgins & Green, 2011), the *I* ^2^ statistic of Higgins (<25% indicates low heterogeneity; 25%–50% moderate heterogeneity; and >50% large heterogeneity) and its *p* value (*p* < .1 indicated large heterogeneity) (Higgins et al., 2002, 2003). We performed an overall meta‐analysis to assess whether body verticality, measured using the SPV test, is altered in patients with somatosensory disturbances, including neurologic and spine diseases, compared to healthy controls.

### 2.7 Subgroup analyses

Second, we performed different subgroup analyses according to specific conditions: (1) According to the plane in which SPV was made, we identified that this test was made in the roll plane and pitch plane. (2) According to the specific pathology assessed in each study included in the review, we identified 2 subgroups (stroke and other CNSD). (3) In the stroke subgroup, we identified different subgroups: stroke without pusher and SN, stroke with pusher, stroke with SN, and stroke with pusher and SN. In all these subgroups, SPV was assessed in the roll and pitch planes.

## 3 RESULTS

### 3.1 Study selection

**FIGURE 1:**
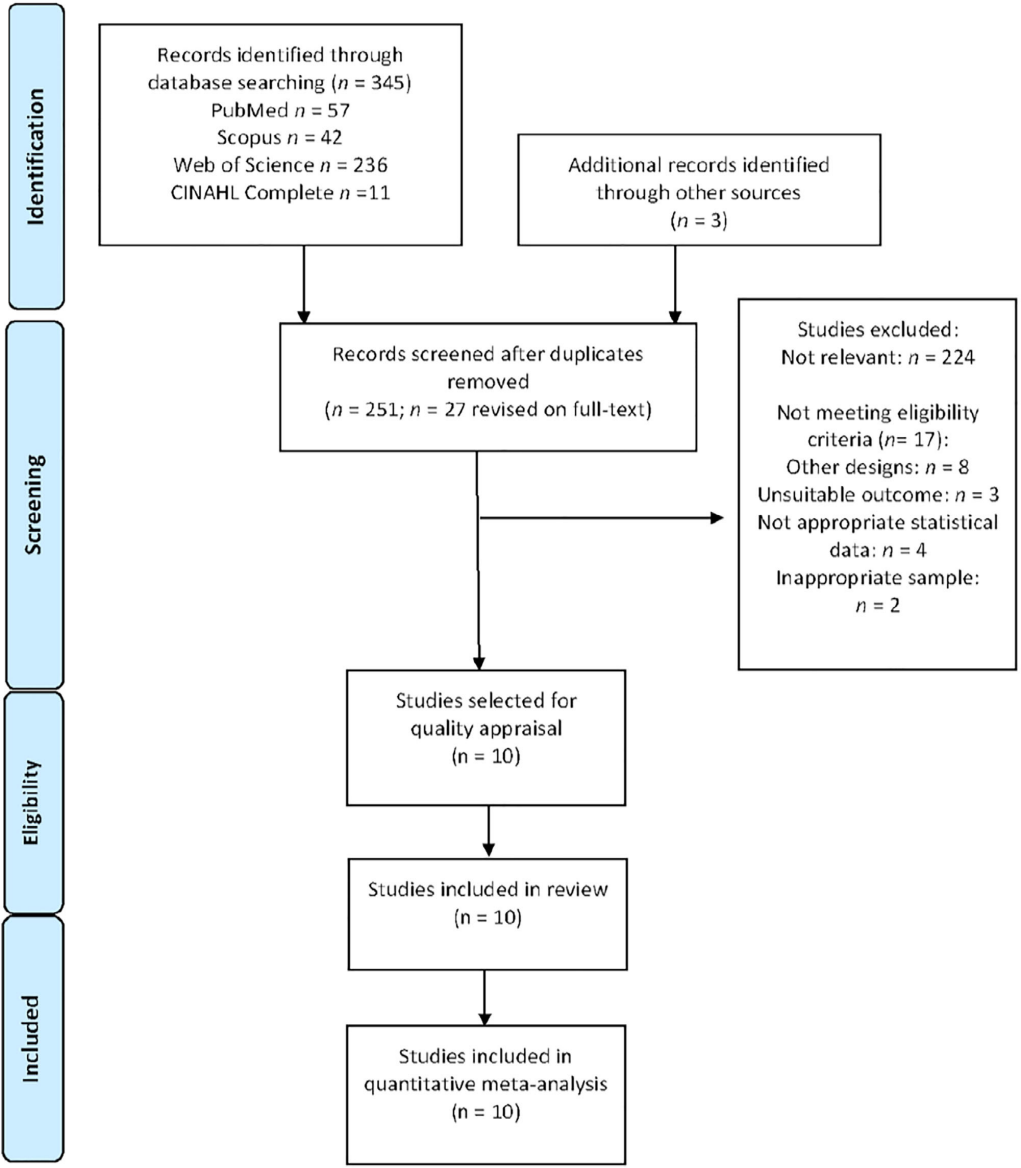
Flow diagram Preferred Reporting Items for Systematic Reviews and Meta‐Analyses (PRISMA) of the study selection process.

Three hundred and forty‐five records were initially retrieved from the health databases (*n* = 57 from PubMed, *n* = 42 from Scopus, *n* = 236 from WOS, and *n* = 11 from CINAHL Complete) and three from other sources. After removing duplicates (*n* = 97), 251 references were screened by title/abstract, and 224 records were excluded for having no relevance to the topic. Twenty‐seven studies underwent full‐text review for eligibility, and 17 studies were deleted for not meeting the inclusion criteria (reasons provided in Figure 1). Finally, 10 observational studies (Anastasopoulos et al., 1999; Bergmann et al., 2016; Fukata et al., 2020a; Joassin et al., 2010; Mansfield et al., 2015; Mazibrada et al., 2008; Pérennou et al., 1998, 2008; Proctor et al., 1964; Selge et al., 2016) were included in the present review. The PRISMA flow chart shown in Figure 1 displays the study selection process.

### 3.2 Characteristics of included studies

The 10 studies included in this meta‐analysis reported 16 independent comparisons. These studies provided data from 390 participants (mean age of 59.30 ± 11.41 years old, 66.8% men and 33.2% female). The exposed group comprised 225 patients (mean age of 61.24 ± 11.16 years old, 66% men and 34% female) who were compared to 165 healthy controls (mean age of 57.1 ± 12.32 years old, 67.8% men and 32.2% female). In the exposed group, 10 studies provided data from patients diagnosed with CNSD (Anastasopoulos et al., 1999; Bergmann et al., 2016; Fukata et al., 2020a; Joassin et al., 2010; Mansfield et al., 2015; Mazibrada et al., 2008; Pérennou et al., 1998, 2008; Proctor et al., 1964; Selge et al., 2016) as stroke (Bergmann et al., 2016; Fukata et al., 2020a; Mansfield et al., 2015; Pérennou et al., 1998, 2008), spinal cord injuries (Joassin et al., 2010; Mazibrada et al., 2008), Parkinson's disease (Proctor et al., 1964), idiopathic hydrocephalus (Selge et al., 2016), and 1 study that included different CNSD (Anastasopoulos et al., 1999). In patients diagnosed with stroke, we identified patients with PS, patients without PS or unilateral SN, patients with unilateral SN, and patients with PS and unilateral SN.

All subjects included in this meta‐analysis were in the chronic phase of the disease. Body verticality perception in all studies was assessed using the SPV test in an upright sitting position. All included studies assessed SPV in the roll plane, and in two of these studies (Bergmann et al., 2016; Selge et al., 2016), SPV was also assessed in the pitch plane. Table 2 shows the primary characteristics of the studies included in this review.

**TABLE 2:** Main characteristics of the studies included in the review.

Exposed group								Healthy controls			SPV test	
	Country	*K*	*N*_s_	*N*_e_	Mean age	Sex (M/F)	Pathology	*N*_c_	Mean age	Sex (M/F)	Plane	Position
Anastasopoulos et al. (1999)	Greece	1	38	18	NR	NR	Neurological mix	20	50.2	NR	Roll	Sitting
Bergmann et al. (2016)	Germany	4	28	8	72.5	(3/5)	Stroke + pusher syndrome	10	70.5	(6/4)	Roll/Pitch	Standing
				10	71.1	(8/2)	Stroke without pusher syndrome				Roll/Pitch	Standing
Fukata et al. (2020a)	Japan	4	58	11	70.1	(5/6)	Stroke + pusher syndrome + unilateral spatial neglect	15	67	(9/6)	Roll	Sitting
				10	66.3	(5/5)	Stroke + pusher syndrome				Roll	Sitting
				10	63.9	(5/5)	Stroke + unilateral spatial neglect				Roll	Sitting
				12	65.4	(6/6)	Stroke without pusher syndrome or unilateral spatial neglect				Roll	Sitting
Joassin et al. (2010)	France	1	27	14	41.6	(12/2)	Spinal cord injuries	13	39.2	(11/2)	Roll	Sitting
Mansfield et al. (2015)	Canada	2	24	7	61.85	(6/1)	Stroke + pusher syndrome	10	65.3	(6/4)	Roll	Sitting
				7	69.57	(5/2)	Stroke without pusher syndrome				Roll	Sitting
Mazibrada et al. (2008)	United Kingdom	1	23	3	34	(3/0)	Spinal cord injuries	20	42	(12/8)	Roll	Sitting
Pérennou et al. (1998)	France	1	36	22	58.3	(16/6)	Stroke without pusher syndrome	14	54.7	(9/5)	Roll	Sitting
Perennou et al. (2008)	France	2	68	29	58	NR	Stroke without pusher syndrome	33	48.8	(22/11)	Roll	Sitting
				6	62.7	NR	Stroke + pusher syndrome				Roll	Sitting
Proctor et al. (1964)	United States	1	58	38	52.3	NR	Parkinson disease	20	51	NR	Roll	Sitting
Selge et al. (2016)	Germany	2	30	20	71	(15/5)	Idiopathic hydrocephalus	10	75	(9/1)	Roll/Pitch	Sitting

Abbreviations: F, female; *K*, number of comparisons; M, male; *N*_c_, number of healthy controls in each study; *N*_e_, number of exposed in each study; *N*_s_, total sample size; SPV, subjective postural vertical.

### 3.3 Methodological quality assessment (Newcastle–Ottawa scale scoring)

The methodological quality of the observational studies included in the present review, as assessed by the NOS (Table 3), was moderate (mean quality: 4.3 ± 1.63 points). Four studies (Anastasopoulos et al., 1999; Bergmann et al., 2016; Joassin et al., 2010; Pérennou et al., 1998) were of low quality (between 1 and 3 stars); five studies of moderate quality (Fukata et al., 2020a; Mazibrada et al., 2008; Pérennou et al., 2008; Proctor et al., 1964; Selge et al., 2016) (between 4 and 6 stars), and one study of high quality (between 7 and 9 stars) (Mansfield et al., 2015). The included studies exhibited a possible risk of selection bias due to low scores obtained by exposed or nonexposed S1–S4 items. Table 3 summarizes the NOS rating for selection, comparability, and exposure/outcome of the selected studies.

**TABLE 3:** Newcastle–Ottawa scale (NOS) score for the methodological quality of the studies included in the review.

Study	S1	S2	S3	S4	C1	E1	E2	E3	Total	Quality
Anastasopoulos et al. (1999)	–	–	–	–	*	*	–	*	3	Low
Bergmann et al. (2016)	–	–	–	–	*	*	–	–	3	Low
Fukata et al. (2020a)	*	*	–	*	*	*	–	*	6	Moderate
Joassin et al. (2010)	–	–	–	*	*	–	–	*	3	Low
Mansfield et al. (2015)	–	*	*	*	*	*	–	*	7	High
Mazibrada et al. (2008)	–	–	–	*	*	*	–	*	4	Moderate
Pérennou et al. (1998)	–	–	–	–	*	–	–	*	2	Low
Perennou et al. (2008)	–	*	–	*	*	*	*	*	6	Moderate
Proctor et al. (1964)	–	*	–	*	*	*	–	*	5	Moderate
Selge et al. (2016)	–	*	–	–	*	*	–	*	4	Moderate

*Note*: Each study can be awarded a maximum of one star for each numbered item within the selection (S) and exposure (E) categories. A maximum of two stars can be given for comparability (C). S1 = adequate case definition; S2 = representativeness of the cases; S3 = selection of controls; S4 = definition of controls; C1 = comparability of cases and controls; E1 = ascertainment of exposure; E2 = same method of ascertainment for cases and controls; E3 = non‐response rate.

### 3.4 Meta‐analysis of misperception of SPV in patients with central nervous system diseases

Ten studies (Anastasopoulos et al., 1999; Bergmann et al., 2016; Fukata et al., 2020a; Joassin et al., 2010; Mansfield et al., 2015; Mazibrada et al., 2008; Pérennou et al., 1998, 2008; Proctor et al., 1964; Selge et al., 2016) with 16 independent comparisons provided data to assess the misperception of SPV in patients with CNSD (Table 4).

**TABLE 4:** Main findings of the meta‐analysis.

Disease	Plane	Effect size				Publication bias			Heterogeneity		
		*K*	SMD	95% CI	*p*‐Value	Funnel plot (Egger *p*‐value)	Trim‐and‐Fill		*Q*‐test	*I* ^2^ (%)	*p‐*Value
							Adj SMD	% of var			
**Central nervous system diseases**	Roll	16	1.05	.84–1.25	<.001	.49	1.36	30	19.61	23.5	.07
	Pitch	3	1.03	.51–1.55	<.001	.11	1.03	0	.09	0	.95
**Stroke**	Roll	11	1.12	.41–1.83	.002	.37	1.43	28	13	23	.22
	Pitch	2	1.09	.41–1.78	.002	NP	NP	NP	.001	0	.97
**Others CNS Diseases**	Roll	5	.87	.32–1.42	.002	.24	.94	8	3.38	0	.49
	Pitch	1	.94	.14–1.73	.021	NP	NP	NP	NP	NP	NP

Abbreviations: 95% CI, 95% confidence interval; Adj, adjusted; *I* ^2^, degree of inconsistency; *K*, number of comparisons; NP, not possible to calculate; SMD, standardized mean difference.

**FIGURE 2:**
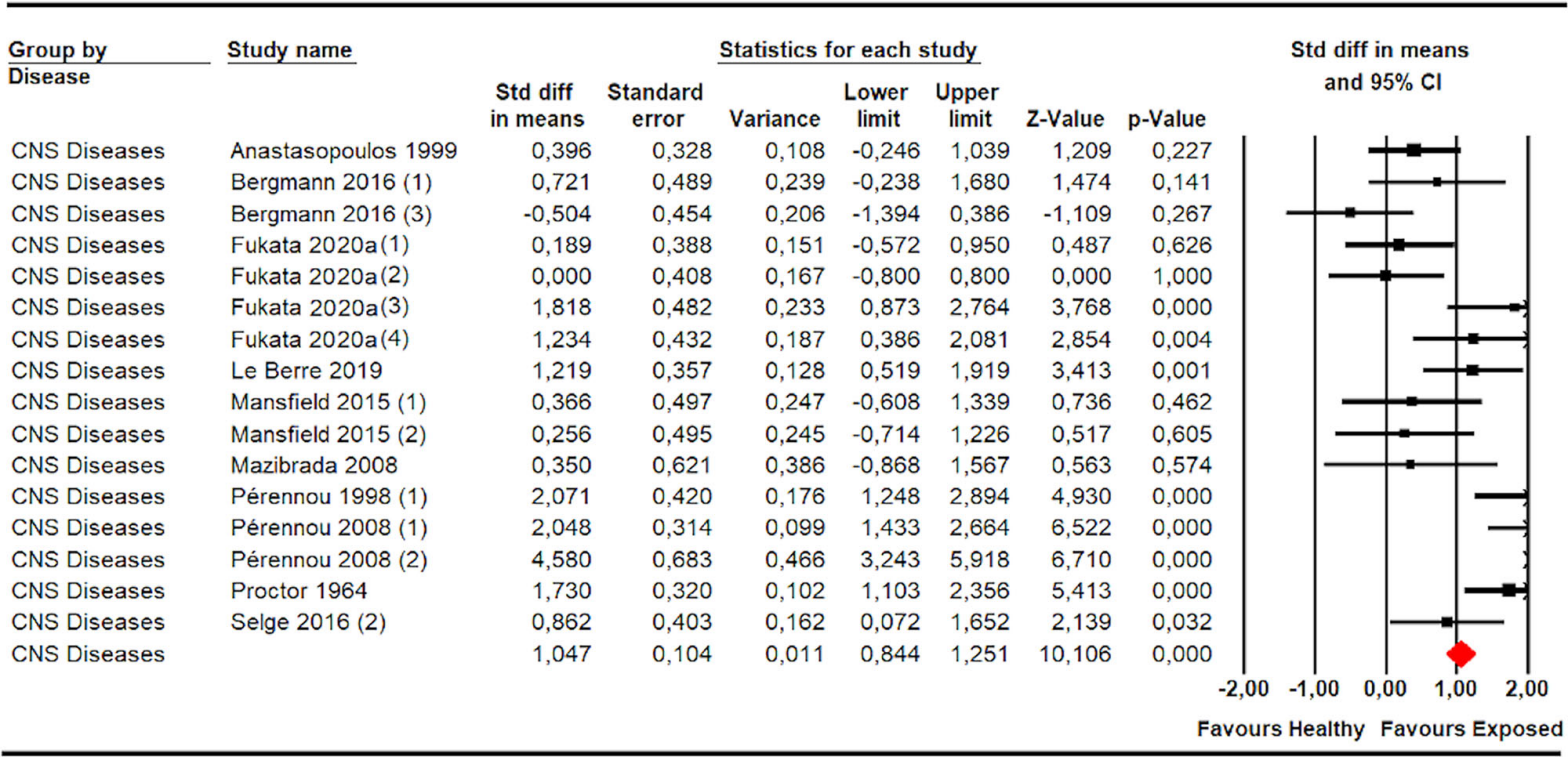
Forest plot of subjective postural vertical in pitch plane in central nervous system diseases (roll plane).

**FIGURE 3:**
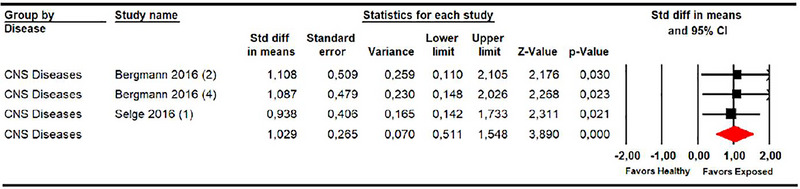
Forest plot of subjective postural vertical in pitch plane in central nervous system diseases (pitch plane).

Patients diagnosed with CNSD displayed a large misperception of SPV in the roll (SMD 1.05; 95% CI .84–1.25; *p* < .001) (Anastasopoulos et al., 1999; Bergmann et al., 2016; Fukata et al., 2020a) (Figure 2; Joassin et al., 2010; Mansfield et al., 2015; Mazibrada et al., 2008; Pérennou et al., 1998, 2008; Proctor et al., 1964; Selge et al., 2016) and pitch planes (SMD 1.03; 95% CI .51–1.55; *p* < .001) (Bergmann et al., 2016; Selge et al., 2016) (Figure 3) compared to healthy controls. The only risk of publication bias was found in the assessment of SPV in the roll plane in patients with CNSD (Egger *p* .49 and 30% change after trim‐and‐fill estimation) with low–moderate heterogeneity (*I* ^2^ 23.5%; *Q* 19.6, df = 15; *p* .07). The precision level was low in all subgroups.

**FIGURE 4:**
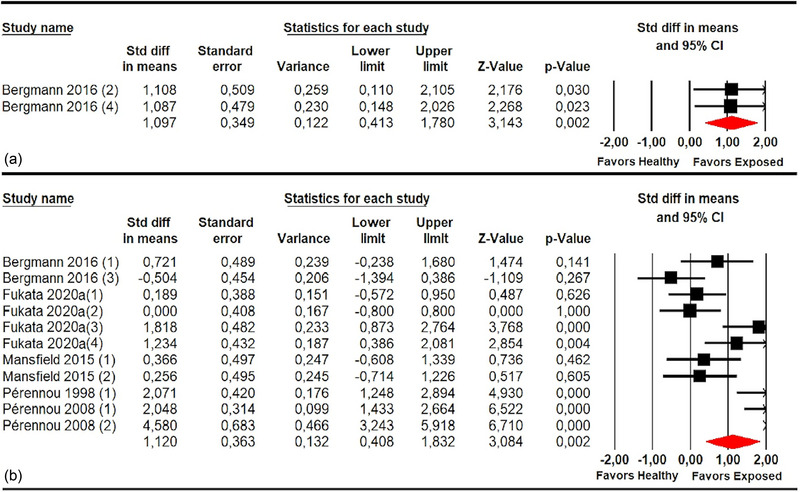
Forest plot of meta‐analyses of subjective postural vertical in pitch (a) and in roll plane (b) in stroke.

In CNSD patients, we differentiated between those diagnosed with stroke (Bergmann et al., 2016; Fukata et al., 2020a; Mansfield et al., 2015; Pérennou et al., 1998, 2008) and other CNSD, such as spinal cord injuries (Joassin et al., 2010; Mazibrada et al., 2008), Parkinson's disease (Proctor et al., 1964), and idiopathic hydrocephalus (Selge et al., 2016) (Table 4). Five studies (Bergmann et al., 2016; Fukata et al., 2020a; Mansfield et al., 2015; Pérennou et al., 1998, 2008) with 11 independent comparisons provided data from patients with stroke in which SPV was assessed in the roll plane, and one study provided 2 independent comparisons for the pitch plane (Bergmann et al., 2016). Our findings showed a misperception of SPV in roll (SMD 1.12; 95% CI .41–1.83; *p* .002) and pitch planes (SMD 1.09; 95% CI .41–1.83; *p* .002) in patients with stroke compared to healthy controls (Figure 4). The risk of publication bias (Egger *p* .37 and 28% of variation after trim‐and‐fill estimation) and low heterogeneity (*I* ^2^ 23%; *Q* 13, df = 10; *p* .22) must be considered in the assessment of SPV in the roll plane. The precision level was also low.

**FIGURE 5:**
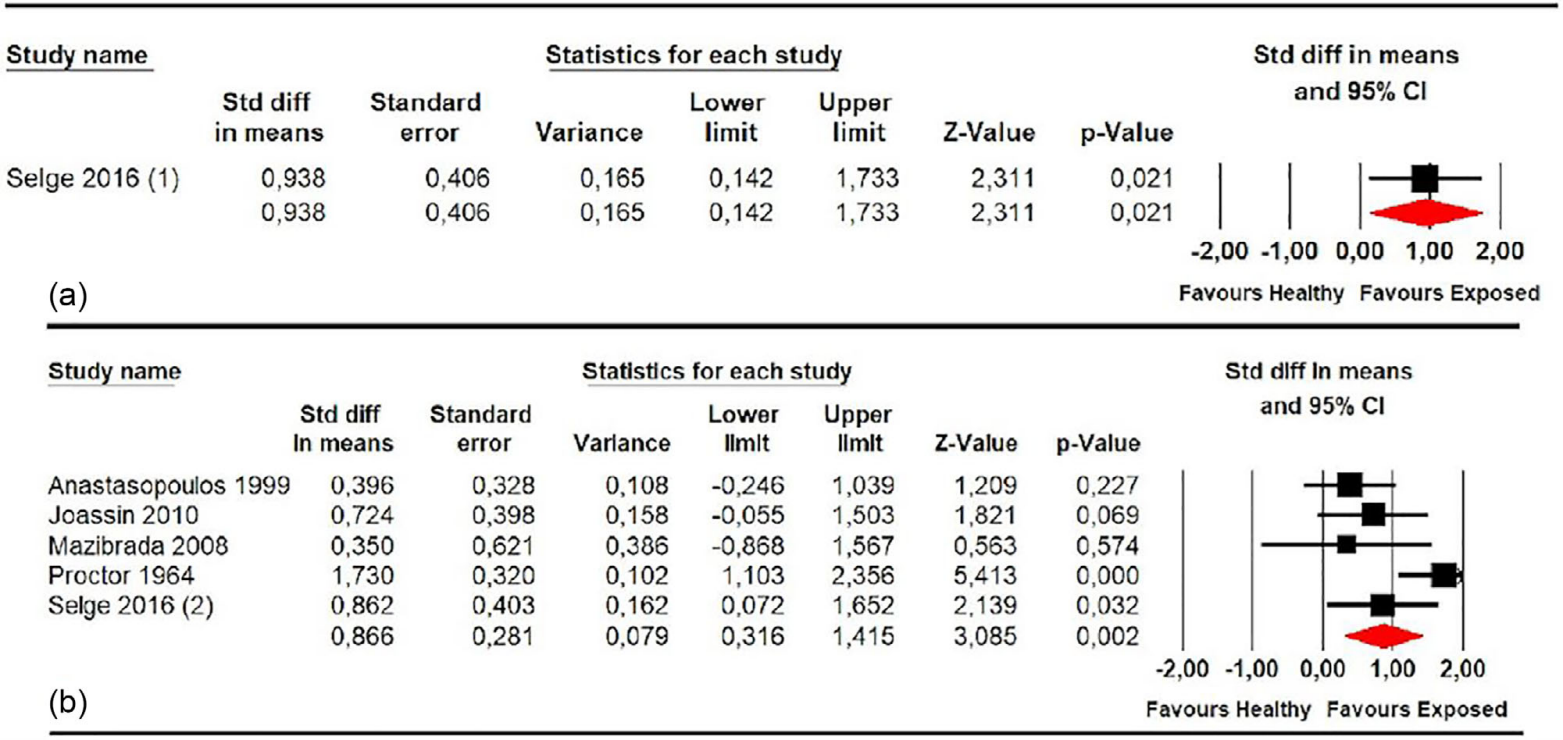
Forest plot of meta‐analyses of subjective postural vertical in pitch (a) and in roll plane (b) in others central nervous system (CNS) diseases.

Additionally, five studies (Anastasopoulos et al., 1999; Joassin et al., 2010; Mazibrada et al., 2008; Proctor et al., 1964; Selge et al., 2016) with five independent comparisons provided data from patients with other CNSD that assessed SPV in the roll plane, and one study (Selge et al., 2016) provided one independent comparison for the pitch plane. Our results revealed a misperception of SPV in these patients in the roll (SMD .87; 95% CI .32–1.42; *p* .002) and pitch planes (SMD .94; 95% CI .14–1.73; *p* .021) compared to healthy subjects (Figure 5, Table 4). A low risk of publication bias was identified in the roll plane analysis (Egger *p* .24 and 8% of variation with the trim‐and‐fill method). The precision level was low in both subgroups.

### 3.5 Meta‐analysis of misperception of SPV in stroke subgroups

**FIGURE 6:**
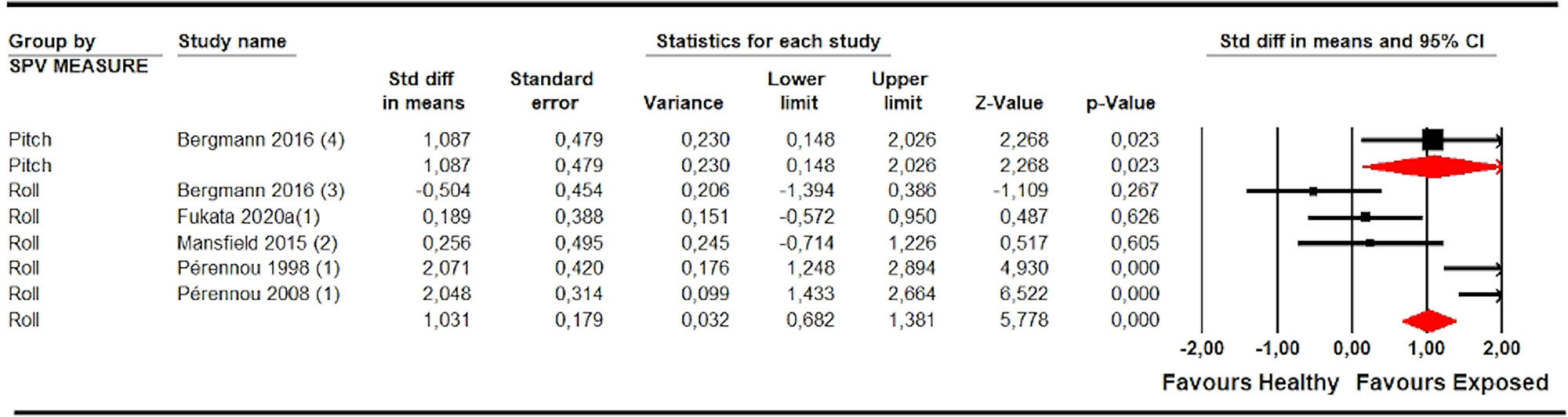
Forest plot of meta‐analyses of subjective postural vertical in pitch and in roll plane in stroke subgroups without pusher and without unilateral spatial neglect.

Misperception of SPV was assessed, more specifically, in patients with stroke with different associated syndromes (Table 5). First, five studies (Bergmann et al., 2016; Fukata et al., 2020a; Mansfield et al., 2015; Pérennou et al., 1998, 2008) with five independent comparisons provided data to assess the misperception of SPV in patients without PS and without SN in the roll plane and one study (Bergmann et al., 2016) with one independent comparison in the pitch plane. A misperception of SPV was observed in these patients in roll (SMD 1.03; 95% CI .68–1.38; *p* < .001) and in the pitch plane (SMD 1.09; 95% CI .15–2.03; *p* .023), with respect to healthy subjects (Figure 6 and Table 5).

**TABLE 5:** Findings of the meta‐analysis in specific stroke subgroups.

Disease	Plane	Effect size				Publication bias			Heterogeneity		
		*K*	SMD	95% CI	*p*‐Value	Funnel plot (Egger *p*‐value)	Trim‐and‐Fill		*Q*‐test	*I* ^2^ (%)	*p*‐Value
							Adj SMD	% of var			
**Stroke without pusher and unilateral spatial neglect**	Roll	5	1.03	.68–1.38	<.001	.22	1.03	0	3.83	0	.42
	Pitch	1	1.09	.15–2.03	.023	NP	NP	NP	NP	NP	NP
**Stroke with pusher**	Roll	4	1.51	1–2.02	<.001	.27	1.51	0	3.96	24.9	.25
	Pitch	1	1.11	.11–2.1	.03	NP	NP	NP	NP	NP	NP
**Stroke with pusher and unilateral spatial neglect**	Roll	1	0	−.8 to .8	1	NP	NP	NP	NP	NP	NP
**Stroke with unilateral spatial neglect**	Roll	1	1.23	.39–2.08	.004	NP	NP	NP	NP	NP	NP

Abbreviations: 95% CI, 95% confidence interval; Adj, adjusted; *I* ^2^, degree of inconsistency; *K*, number of comparisons; NP, not possible to calculate; SMD, standardized mean difference.

**FIGURE 7:**
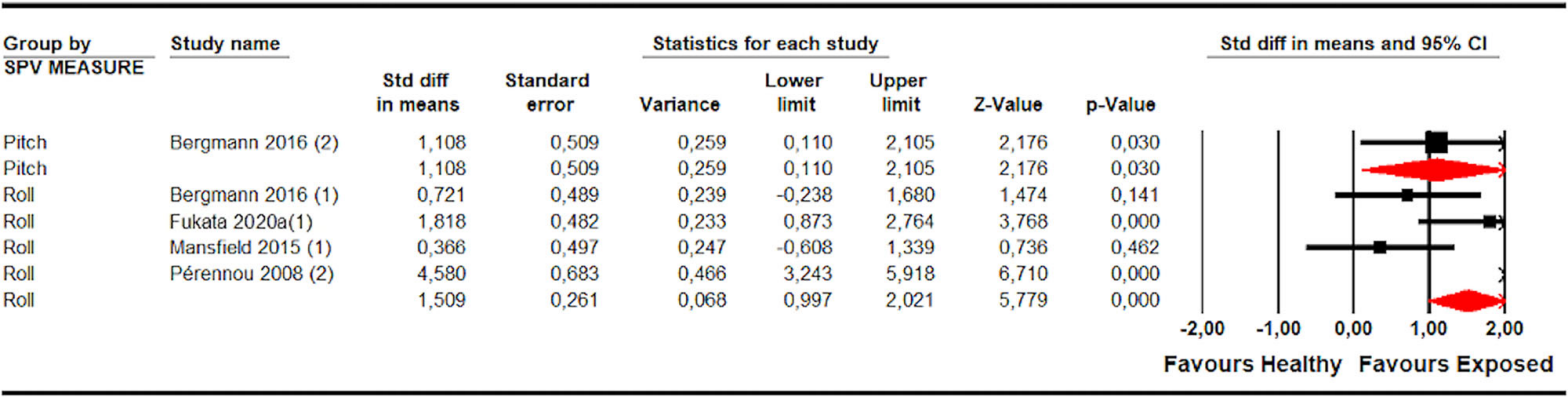
Forest plot of meta‐analyses of subjective postural vertical in pitch and in roll plane in stroke subgroups with pusher.

Second, four studies (Bergmann et al., 2016; Fukata et al., 2020a; Mansfield et al., 2015; Pérennou et al., 2008) with four independent comparisons provided data to assess the misperception of SPV in patients with PS in roll and one study (Bergmann et al., 2016) with one independent comparison in the pitch plane. These patients exhibited an alteration in SPV in the roll (SMD 1.51; 95% CI 1–2.02; *p* < .001) and pitch planes (SMD 1.11; 95% CI .11–2.1; *p* .03) compared to healthy controls (Figure 7 and Table 5).

**FIGURE 8:**
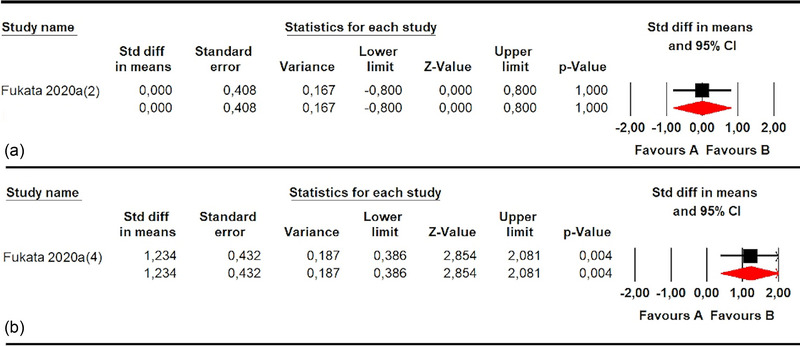
Forest plot of meta‐analyses of subjective postural vertical in roll plane in stroke subgroups with unilateral spatial neglect (a) and in roll plane in stroke subgroups with pusher and unilateral spatial neglect (b).

One study (Fukata et al., 2020a) with one independent comparison assessed SPV in stroke patients with unilateral SN and showed a misperception of SPV (SMD 1.23; 95% CI .39–2.08; *p* .004) compared to healthy subjects (Figure 8 and Table 5). Finally, one study (Fukata et al., 2020a) provided one independent comparison of reported data to assess the SPV in patients with unilateral SN in the roll plane, but no statistically significant differences (SMD 0; 95% CI −.8 to .8; *p* 1) were found between the groups (Figure 8 and Table 5).

## 4 DISCUSSION

The present systematic review and meta‐analysis was performed to analyze possible alterations of SPV in patients with CNSD. The results obtained have demonstrated altered SPV in both the roll and the pitch planes. In patients with stroke, the alteration was more pronounced in the roll plane than in the pitch plane, especially in those with PS, followed by those with PS combined with hemineglect.

This meta‐analysis has showed an alteration of the SPV in both planes in subjects with CNSD. One of the possible causes of these results could be the probable alteration of the somatosensory system, which is key for a suitable estimation of the SPV (Dakin & Rosenberg, 2018; Nakamura et al., 2020). It has been shown that the SPV test primarily evaluates somatosensory and vestibular contributions to vestibular patterns, although the latter only acts as a modulator, since it has been observed that voluntary modification of vestibular afferences in healthy subjects does not alter SPV (Nakamura et al., 2020). Therefore, a marked impairment of SPV in these subjects may be due to an alteration of the inputs of proprioceptive signals or its processing. This hypothesis is consistent with previous findings observed in subjects with stroke, having found more significant alterations of the SPV in cases with greater involvement of the somatosensory system (Saeys et al., 2012). Moreover, it seems that patients with stroke who are also affected by PS may present greater involvement of the SPV (Fukata et al., 2020a, 2020b; Mansfield et al., 2015), which may be explained by reduced sensitivity to somatosensory graviception among patients with PS (Fukata et al., 2020a).

Test parameters should also be considered when SPV impairments are analyzed. It has been observed a greater variability and higher importance of visual inputs during sitting than in standing position (Bergmann et al., 2020), which may suggest different strategies for verticality estimation while sitting and while standing (Bergmann et al., 2020), as well as a reduction of the somatosensory inputs. This fact may be explained by the greater amount of somatosensory information from the hip, knee, and foot afferences, which is diminished when patients perform the test in a sitting. Conversely, although conclusive results regarding this issue have not yet been achieved, the importance of the initial position of the patient during SPV evaluation has also been hypothesized. In patients with PS, when the test started from the healthy side, a greater SPV misperception was appreciated (Fukata et al., 2020a), although these results disagree with previous research, in which the opposite result was obtained (Karnath & Broetz, 2003). Another factor that should be considered is fixing the head to the backrest, as worse results have been observed in patients who perform the test with their head fixed instead of being left without mobility restrictions. The provision of slight neck proprioceptive adjustments during the test enables compensations and, hence, a good verticality perception (Ashish et al., 2017; Chang et al., 2019). Performing the SPV test with the head fixed produces a loss of proprioceptive information in the same way that occurs when the test is performed in a sitting position. All the above highlight the primary role that the somatosensory system plays in verticality pattern construction from posture and the need to establish a solid protocol to perform the SPV test.

Despite interest in the results achieved in the present work, this review has several limitations. First, the low number of studies included in each meta‐analysis and the low sample size of each study might make it difficult to generalize these results. Second, the methodological quality of the included studies may lead to selection bias. In addition, the risk of publication bias found in some meta‐analyses may underestimate the original pooled effect. In future research, it would be advisable to perform studies with a larger sample size to avoid possible selection bias. Likewise, it would be interesting in future research to establish a solid protocol to perform the SPV test, allowing us to reach conclusions that can be generalized beyond of SPV evaluation parameters. Progress in the aforementioned lines of research would be of interest for the development of new treatments that could improve the perception of SPV in these patients and, therefore, achieve a possible improvement in their clinical outcomes.

## 5 CONCLUSION

In light of the results of this review, it can be concluded that patients with CNSD have a misperception of body verticality in both the roll and pitch planes. Although the results are statistically significant, the assessment of the deviation in the pitch plane is more doubtful due to the paucity of data with very few studies included in the review. More studies would be needed to increase the power of the analysis and to be able to analyze other important variables such as the effect of the passage of time and the stage of the patients in order to be able to analyze the evolution of the misperception of verticality in these patients.

## AUTHOR CONTRIBUTIONS

*Conceptualization; software; formal analysis; writing—original draft; writing—review and editing*: Esteban Obrero‐Gaitán. *Conceptualization; investigation; validation; writing—original draft*: David Fuentes‐Núñez. *Conceptualization; investigation; visualization; formal analysis*: María del Moral‐García. *Visualization; validation; methodology; supervision*: María del Carmen López‐Ruiz. *Conceptualization; validation; investigation; writing—original draft; writing—review and editing*: Daniel Rodríguez‐Almagro. *Conceptualization; methodology; writing—original draft; writing—review and editing; visualization; supervision; project administration*: Rafael Lomas‐Vega. All authors have read and agreed to the published version of the manuscript.

## CONFLICT OF INTEREST STATEMENT

The authors declare that there are no conflicts of interest.

## FUNDING INFORMATION

This research received no specific grant from any funding agency in the public, commercial, or not‐for‐profit sectors.

### PEER REVIEW

The peer review history for this article is available at https://publons.com/publon/10.1002/brb3.3496.

## Data Availability

The data that support the findings of this study are available from the corresponding author upon reasonable request.
